# Profiling post-translational modifications of histones in human monocyte-derived macrophages

**DOI:** 10.1186/s12953-015-0080-7

**Published:** 2015-09-24

**Authors:** Pawel Olszowy, Maire Rose Donnelly, Chanho Lee, Pawel Ciborowski

**Affiliations:** Department of Pharmacology and Experimental Neuroscience, University of Nebraska Medical Center, Omaha, NE 68198-5880 USA; Department of Environmental Chemistry and Bioanalytics, Faculty of Chemistry, Nicolaus Copernicus University, Gagarin 7 Street, 87-100 Torun, Poland

**Keywords:** Histones, Post-translational modification, Proteomics, Mass spectrometry, Macrophage, Innate immunity

## Abstract

**Background:**

Histones and their post-translational modifications impact cellular function by acting as key regulators in the maintenance and remodeling of chromatin, thus affecting transcription regulation either positively (activation) or negatively (repression). In this study we describe a comprehensive, bottom-up proteomics approach to profiling post-translational modifications (acetylation, mono-, di- and tri-methylation, phosphorylation, biotinylation, ubiquitination, citrullination and ADP-ribosylation) in human macrophages, which are primary cells of the innate immune system. As our knowledge expands, it becomes more evident that macrophages are a heterogeneous population with potentially subtle differences in their responses to various stimuli driven by highly complex epigenetic regulatory mechanisms.

**Methods:**

To profile post-translational modifications (PTMs) of histones in macrophages we used two platforms of liquid chromatography and mass spectrometry. One platform was based on Sciex5600 TripleTof and the second one was based on VelosPro Orbitrap Elite ETD mass spectrometers.

**Results:**

We provide side-by-side comparison of profiling using two mass spectrometric platforms, ion trap and qTOF, coupled with the application of collisional induced and electron transfer dissociation. We show for the first time methylation of a His residue in macrophages and demonstrate differences in histone PTMs between those currently reported for macrophage cell lines and what we identified in primary cells. We have found a relatively low level of histone PTMs in differentiated but resting human primary monocyte derived macrophages.

**Conclusions:**

This study is the first comprehensive profiling of histone PTMs in primary human MDM. Our study implies that epigenetic regulatory mechanisms operative in transformed cell lines and primary cells are overlapping to a limited extent. Our mass spectrometric approach provides groundwork for the investigation of how histone PTMs contribute to epigenetic regulation in primary human macrophages.

**Electronic supplementary material:**

The online version of this article (doi:10.1186/s12953-015-0080-7) contains supplementary material, which is available to authorized users.

## Introduction

Post-translational modifications (PTM) of histones comprise the exceptionally complex system of epigenetic regulation (“histone code”) of cellular functions. The major source of complexity originates from multiple combinations of homogenous and heterogeneous PTMs. These modifications include acetylation, mono-, di- and tri-methylation, phosphorylation, ubiquitination, ADP-ribosylation, biotinylation and citrullination [1–3]. Nevertheless, histone PTMs are a universal mechanism of regulation in the sense that they are also involved in normal (healthy) cellular processes [4, 5]. When cells are exposed to various external stimuli, i.e., toxins or toxic substances, malignant transformation [6], bacterial [7] or viral infections [8], the histone code changes, resulting in phenotypic (functional) adaptations of the cells, which may allow them to perform desired tasks or lead to a pathological outcome [6, 9]. Because of the universality of the histone code, it is necessary to establish a pattern of histone PTMs in cells that are considered to be in their unaffected and resting physiological state before any further manipulations are imposed. Therefore, our goal in this study is to identify such a pattern in human monocyte-derived macrophages (MDM).

Mononuclear phagocytes (MPs: monocytes, dendritic cells, tissue macrophages, connective tissue histiocytes, Langerhans cells of skin, Kupffer cells of liver, and microglial cells of brain) comprise the principal elements of the innate immune system [10–13]. Monocytes differentiate to macrophages and dendritic cells, thereby replenishing these cells under normal states, as well as respond quickly to pro-inflammatory signals, infections or toxic insults. They are produced in the bone marrow from monoblasts, circulate in the bloodstream for a relatively short period of time and eventually settle in tissues throughout the body, where they differentiate into tissue macrophages. Macrophages maintain homeostasis by eliminating microbial pathogens and clearing debris, all while responding to a variety of environmental stimuli. Such stimuli leads to a variety of responses including cell mobility, phagocytosis, antigen presentation, up-regulation of secreted pro-inflammatory cytokines, reactive oxygen species, quinolinic acid, glutamate, arachidonic acid, and its metabolites etc., thus contributing to the overall response of the innate immune system [14–16]. Abnormalities in epigenetic regulatory processes will eventually lead to damaging consequences in the function of macrophages and their role as the first line of immune defense. Despite decades of research, the mechanisms of how microbes evade immune-surveillance as well as the effect of other factors such as exposure to toxins, illicit and other drugs etc., which contribute to a wide range of impairments of the innate immune system, are still far from being understood.

Most of the work done on investigating epigenetic mechanisms resulting from histone PTMs in macrophages has been performed predominantly using three cell lines: U937, THP1 and murine RAW264.7 [17–22]. The use of cell lines is common due to the fact that using primary human cells as a model has experimental limitations, such as inherent donor-to-donor variability and relative resistance of primary macrophages to transfection, a method ubiquitously used for mechanistic studies. Nevertheless, MDMs are an attractive model as they are more reflective of the *in vivo* response than transformed cell lines. The intent of our study design was to establish a baseline histone code for MDMs under the physiological conditions of a resting cell as the first step into deeper investigations of epigenetic regulation occurring under various pathological conditions. In this work, we present the first comprehensive approach in profiling PTMs of histones isolated from human MDMs obtained from healthy individuals.

Recently, mass spectrometry has been widely utilized to profile PTMs of histones, providing direct information on the site and types of modifications. In this study, we used LTQ XL Orbitrap ETD (Thermo Scientific, Inc.) and 5600 qTof/TripleTOF (ABSciex, Inc.) with two methods of data acquisition utilizing collision induced dissociation (CID) and electron transfer dissociation (ETD) to yield complementary identifications of PTMs. Although significant experimental work in mass spectrometric analysis of histone PTMs has been done, our data indicates that some modifications identified in transformed cell lines may not exist in primary cells, thus building a foundation for further investigations of epigenetic gene regulation of MPs using primary human cells.

## Materials and methods

### Reagents

Acetonitrile (ACN), methanol, water (HPLC-gradient grade), 0.1 % formic acid (FA) in water (*v*/*v*), and 0.1 % formic acid in acetonitrile (Optima® LC/MS grade) were purchased from Fisher Scientific (Fair Lawn, NJ, USA). Acetic acid (99.7 + %, A.C.S. reagent), trifluoroacetic acid (TFA, ReagentPlus®, 99 %), ammonium bicarbonate (ReagentPlus®, 99 %) and iodoacetamide (IAA, Sigma Ultra) were purchased from Sigma-Aldrich (St. Louis, MO, USA). Dithiothreitol (DTT, molecular grade) and modified trypsin (sequencing grade) were purchased from Promega Corporation (Madison, WI, USA).

### Sample preparation

Primary human monocytes were obtained by leukophoresis from donors who were seronegative for HIV-1, HIV-2, and hepatitis B virus. Peripheral Blood Mononuclear Cells (PBMC) from HIV-1, HIV-2 in full compliance and approval of the University of Nebraska Medical Center Institution Review Board. Monocytes were purified using counter-current centrifugal elutriation and cultured at a final concentration of 1 million per mL in macrophage serum-free medium (M-SFM) purchased from Invitrogen® (Carlsbad, CA, USA). M-SFM was supplemented with a 1 % HEPES buffer (4-(2-hydroxyethyl)-1-piperazineethanesulfonic acid) also purchased from Invitrogen® (Grand Island, NY, USA); 1 % Nutridoma was purchased from Roche Diagnostics GmbH (Mannheim, Germany), and Macrophage Colony Stimulating Factor (MCSF) was purchased from PeproTech (Rocky Hill, NJ, USA). Half media exchanges were performed on days 3 and 5 after seeding cells.

Monocyte-derived macrophages (MDM) were harvested on day 7. Proteins were fractionated using two different approaches. In the first fractionation approach, we used a Qproteome Nuclear Protein Kit (QIAGEN®, Hilden, Germany). Each fraction - cytosolic, nuclear, and histone proteins - were quantified using the Pierce® 660 nm Protein Assay in a pre-diluted protein standard of Bovine Serum Albumin (BSA), both from Thermo Scientific (Rockford, IL, USA). The second fractionation approach was acid extraction. Differentiated MDMs were washed twice with ice-cold PBS and harvested with PBS containing 5 mM sodium butyrate. Following centrifugation at 2000 rpm for 5 min at 4 °C, the supernatant was removed and cells were re-suspended in Triton Extraction Buffer (TEB: PBS containing 0.5 % Triton X 100 (*v*/*v*), 2 mM phenylmethylsulfonyl fluoride (PMSF) and 0.02 % (w/v) NaN_3_). Cells were lysed for 10 min with gentle stirring, then centrifuged at 2000 rpm for 10 min at 4 °C and the supernatant was removed and discarded. Cells were washed in half volume of TEB and centrifuged as before. After that, the pellet was re-suspended in 0.2 M HCl and kept overnight (16–18 h) at 4 °C with gentle stirring. Following the overnight stirring, the sample was centrifuged at 2000 rpm for 10 min at 4 °C, the supernatant was removed, and protein concentration was determined as described previously.

The histone fraction isolated from MDMs was further fractionated using a 1D gel electrophoresis system, XCell SureLock™ Mini-Cell, which was purchased from Invitrogen®) (Life Technologies Corporation, Grand Island, NY, USA), and connected with a PowerPac™ basic power supply (Bio-Rad Laboratories Inc., Hercules, CA, USA). For separation of histones, NuPAGE® 4–12 % Bis-Tris Gel 1.0 mm was used (Invitrogen®, Carlsbad, CA, USA). According to the manufacturer’s protocol, sample buffer was prepared by adding 0.25 mL of NuPAGE® LDS sample buffer (4×), 0.1 mL NuPAGE® sample-reducing agent (10×), and 0.65 mL of HPLC-grade water (Fair Lawn, NJ, USA). Samples were re-suspended in sample buffer and heated for 5 min at 95 °C. Seven microliters of SeeBlue® Plus2 pre-stained molecular mass marker (Invitrogen®, Carlsbad, CA, USA) and 5 μg of histones were loaded into the wells of the gel. Fractionation of histones was performed under constant voltage (100 V) for 90 min. After separation, the gel was removed from the cassette and placed in a fixing solution (40 % methanol or ethanol, 7 % acetic acid) for 1 h. After 1 h, the gel was placed overnight in a staining solution of Brilliant Blue G – Colloidal Concentrate Electrophoresis Reagent (Sigma® Life Science, St. Louis, MO, USA). The staining solution was prepared immediately before initiating staining by combining four parts Brilliant Blue G to one part methanol or ethanol. After an overnight treatment in staining solution, the gel was immersed in de-staining solution (10 % acetic acid, 25 % methanol or ethanol) for 60 s, rinsed briefly with 25 % methanol or ethanol, and placed in 25 % methanol or ethanol overnight for further de-staining. The gel was then scanned and bands were excised prior to tryptic digestion.

Gel pieces were washed by placing them into plastic vials with 100 μL of 50 % acetonitrile (ACN) and washed for five minutes on a tilt table. After removing the wash solution, 100 μL of 50 % ACN/50 mM ammonium bicarbonate (NH_4_HCO_3_) was added, and samples were washed for 30 min on a tilt table. After removal of the second wash solution, 100 μL of 50 % ACN/10 mM NH_4_HCO_3_ (Fisher A669-500) was added, and samples were washed for 30 min on a tilt table. Following drying in a SpeedVac, 2 μL (0.1 μg/μL) of modified trypsin were added to the prepared gel pieces in their vials and let to stand for 5–10 min in order to allow the enzyme/buffer solution to be absorbed into the gels. Following this step, an additional 50 μL of 10 mM NH_4_HCO_3_ were added. Gels were incubated in 37 °C for 18 h. Resulting peptides were extracted by shaking at room temperature for 1 h in 100 μL of 0.1 % trifluoroacetic acid in 60 % acetonitrile. This step was repeated twice. Combined washes were then dried and re-dissolved in 0.5 % TFA in water. Samples were cleaned up using C_18_ u-ZipTip® pipette tips (EMD Millipore, Billerica, MA, USA); the manufacturer-recommended protocol was used in this step. Lastly, prior to mass spectrometry analysis, samples were re-dissolved in 7 μL of 0.1 % formic acid in water (*v*/*v*).

### Mass spectrometry

#### Nano-LC-LTQ-Orbitrap

Samples were analyzed using a high resolution mass spectrometry ESI-LC-MS/MS system in a nano-spray configuration (LTQ Orbitrap XL, Thermo Scientific, West Palm Beach, FL, USA) coupled with a nano-liquid chromatography system (TEMPO™ nano MDLC System, AB SCIEX, Framingham, MA, USA) equipped with two alternating peptide traps and a PicoFrit RP-C_18_ column (New Objectives, Woburn, MA, USA). The applied mass spectrometer was tuned by direct infusion of angiotensin and calibrated every 2 to 3 days using standards provided by the manufacturer.

Five microliters of each sample were loaded onto the peptide trap with 98:2 (*v*/*v*) HPLC water with 0.1 % formic acid: ACN with 0.1 % formic acid. The samples were then eluted using a 60-minute linear gradient of 0–60 % of acetonitrile in 0.1 % of formic acid.

The acquisition method was created in a data-dependent mode with one full scan in the Orbitrap, followed by fragmentation of the five most-abundant peaks using CID/ETD and peptide fragmentation in the linear ion trap (LTQ). Resolution of the full scan in the Orbitrap was set to 60,000 m/z with a range from 300 to 2000 Da. Precursor peaks with a minimum signal count of 50,000 were dynamically excluded after two fragmentations for 60 s within a range of +/−10 ppm. Monoisotopic precursor selection (MIPS) was enabled. Charge state rejection was not used, but previously located background peaks were included in a mass rejection list. The collision energy was set to 35 kV using an isolation width of 2 m/z and an activation Q of 0.250.

### chip-LC-5600 TripleTOF

Samples were analyzed using an ESI-LC-MS/MS system in a nano-spray configuration (ABSciex 5600 TripleTOF®, AB SCIEX, Framingham, MA, USA) coupled with an ultra-nano-HPLC with a cHiPLC system (Eksigent, Dublin, CA, USA). Samples were loaded onto a 0.5 mm C_18_ CL 3 μm 120 Å trap column (Eksigent, Dublin, CA, USA), washed with 98:2 HPLC water with 0.1 % formic acid: ACN with 0.1 % formic acid for 10 min and then eluted through a 15 cm C_18_ CK 3 μm 120 Å ChromXP column (Eksigent, Dublin, CA, USA) with 98:2 HPLC water with 0.1 % formic acid: ACN with 0.1 % formic acid using a 60-minute linear gradient of 0–60 % ACN with 0.1 % formic acid. The instrument was calibrated every 5–6 samples using 25 fmol of β-galactosidase standards provided by the manufacturer. The acquisition method was in the data dependent mode with one full scan followed by fragmentation of the 50 most abundant peaks. Precursor peaks with a minimum signal count of 100 were dynamically excluded after two selections for 6 s within a range ± 25 mDa. Charge states other than 2–5 were rejected. Rolling collision energy was used.

### Propionylation

Chemical derivatization of histones by propionylation was done following a protocol published by Garcia et al. [23]. This process was done with 5 μg of the histone fractions collected from MDMs with the QProteome Nuclear Protein kit, as previously described. The procedure consisted of four rounds of propionylation, with two occurring before trypsin digestion and two after. Samples were dried down in a SpeedVac concentrator and reconstituted in 5 μl of 100 mM ammonium bicarbonate (Sigma A6141). Propionylation was then implemented with the following sequence of steps: 1) 2 μl of ammonium hydroxide (Sigma-Aldrich 338818) was added to the samples, 2) 20 μl of fresh propionylation reagent, which is composed of 75 % propionic anhydride (Alfa Aesar A12955) and 25 % D_0_-methanol (Fisher A412P), were added to the samples, mixed by vortex and briefly spun down in a tabletop microcentrifuge, 3) pH was tested with pH indicator strips and readjusted to 8 if necessary with a drop-wise addition of ammonium hydroxide, 4) samples were incubated at 51 °C for 20 min, 5) samples were dried down to 5 μl in a SpeedVac concentrator. The samples were then diluted in 5 μl of 100 mM ammonium bicarbonate and steps 1–5 were repeated once. Next, 50 μl of 100 mM ammonium bicarbonate were added and the samples were incubated overnight at 37 °C with a 1:20 (trypsin: protein) ratio of sequencing-grade modified trypsin (Promega V511A). Acetic acid was added in a drop-wise fashion to decrease the pH to ≤3 and samples were briefly placed in −80 °C to inactivate trypsin. Samples were dried down to 5 μl and steps 1–5 were repeated once. Samples were then diluted in 5 μl of 100 mM ammonium bicarbonate, steps 1–4 were completed a final time, and the resulting peptides were dried down completely in a SpeedVac concentrator.

The first round of sample cleanup was done using Oasis mixed cation-exchange cartridges (Waters; Milford, MA). The previously dried peptides were reconstituted in 1 ml of 0.2 % formic acid. The pH was checked with pH indicator strips and additional formic acid was slowly added as necessary to lower the pH to ≤3. The MCX cartridge was equilibrated by passing 1 ml of 1:1 methanol:water across the cartridge. The sample was applied to the column at a rate of ~1 drop per second. The cartridge was then washed with 1 ml of wash solution, composed of 5 % methanol (Fisher A412P) and 0.1 % formic acid in water. A second wash was done with 1 ml of 100 % methanol (Fisher A412P). Bound peptide was eluted with 1 ml of fresh elution buffer, composed of 50 μl of 28 % NH_4_OH solution and 950 μl of methanol. Eluted peptides were dried down completely in a SpeedVac concentrator. The second round of sample cleanup was done using C_18_ u-ZipTip® pipette tips in accordance to the manufacturer’s protocol (Millipore, Billerica, MA). Peptides were quantified on a NanoDrop2000 (Thermo Scientific, Wilmington, DE) at 280 nm and 1 μg of each sample was used for mass spectrometry analysis.

### Identification of histone PTMs

Searches of PTMs were performed using three algorithms: PEAKS 6.0 (Bioinformatics Solutions Incorporation, Waterloo, Canada), Mascot (Matrix Science, Boston, MA), and ProteinPilot 5.0 (Ab Sciex, Framingham, MA). The search parameters for PEAKS were as follows: 1) variable modifications were carbamidomethylation on Cysteine (C) and oxidation on methionine (M); 2) three missed cleaved trypsin sites were permitted; 3) the mass accuracy for the peptide (MS) was set at 10 ppm and for fragments (MS/MS) at 0.8 Da; 4) false discovery rate (FDR) was set at 1 %; 5) variable posttranslational modifications were acetylation, mono-, di-, and tri-methylation, phosphorylation, biotinylation, ubiquitination, citrullination, and ADP-ribosylation. The search parameters for Mascot were as follows: 1) SwissProt 57.15 database was used; 2) variable modifications were acetyl (Lys), acetyl (N-term), carbamidomethyl (Cys), oxidation (His, Trp), propionyl (Lys), propionyl (N-term) and propionyl (Ser); 3) mass values were set to monoisotopic and the protein mass was unrestricted; 4) the peptide mass tolerance was set to ± 0.8 Da and the fragment mass tolerance was set to ± 0.5 Da; 5) a maximum of 1 missed cleavage was allowed. The search parameters for ProteinPilot were as follows: 1) paragon algorithm 5.0 was used; 2) special factors were acetylation emphasis, propionylation pre-digestion and propionylation post-digestion; 3) identification focus was on biological modifications; 4) the search effort was set to ‘thorough’.

## Results and discussion

### Extraction of histones

Histone octamers, composed of H2A, H2B, H3, and H4, are essential for compacting DNA into a cell’s nuclei. One octamer is used to wrap 146 bp and Histone H1 acts as a linker, wrapping an additional 20 bp, totaling 166 bp per one nucleosome. Considering that the human genome has approximately 3 × 10^9^ bp, one would need approximately 2 × 10^7^ nucleosomes or 1.8 × 10^8^ histone molecules to wrap all the DNA [24], making histones highly abundant proteins.

In this study we used two methods of histone isolation, one using a Qproteome Nuclear Protein Kit and the other using acid extraction. The kit method provides a multistep protocol to isolate nuclei in the initial steps and the insoluble nuclear protein fraction containing histones, is isolated in the very last step. Based on our studies (unpublished data), an average yield of nuclei preparation from MDMs using this protocol is between 15 and 20 %. Protocols used by others also include isolation of nuclei [25–27], which we consider as the major yield-limiting step. The subsequent preparative step of separating soluble nuclear proteins further contributes to sample loss, although to a lesser extent. The yield of histones is, on average, 12 μg from 1.2 × 10^7^ MDM cells. Technical and biological replicates of the histone yield are presented in Table 1, showing a 24 % inter-variability while intra-variability remains below 8 %, indicating quite significant donor-to-donor variability. It is important to note that the human monocytes used in this study are primary cells and cell cultures were normalized once at the beginning by seeding 3 × 10^6^ monocytes per well. MDMs are adherent cells and the final number of adhering and differentiating monocytes during a seven day culture varies between donors, cultures, and plate wells. This certainly contributes to substantial inter-variability. In summary, based on the calculations presented above, we estimate that the protocol, which includes isolation of nuclei, yields a histone preparation of approximately 15 to 20 %.

**Table 1:** Intra- and inter-variability of histone yield^a^

	Donor 1	Donor 2	Donor 3
Prep 1 [μg]	12.44	15.95	10.01
Prep 2 [μg]	10.78	14.91	9.23
Prep 3 [μg]	11.02	15.12	9.62
Intra-variability			
Average [μg]	11.41	15.32	9.62
SD [μg]	0.90	0.55	0.39
RSD [%]	7.86	3.59	4.05
Inter-variability			
Average [μg]	12.12		
SD [μg]	2.92		
RSD [%]	24.07		

^a^Each preparation used one 6-well plate corresponding to 12 × 10^6^ seeded monocytes

Another method of histone preparation is acid extraction. We followed a protocol provided by Abcam, Inc. (http://www.abcam.com/index.html?pageconfig=resource&rid=11410), resulting in approximately four times more yield of total proteins than the Qiagen protocol that isolates nuclei. However, this resulting histone preparation was not as pure compared to the fraction obtained from the Qproteome Nuclear Protein Kit, as evaluated by 1-dimensional gel electrophoresis (data not shown). The purity of the histone fraction may have a less overall effect when bottom-up analysis is preceded by 1-DE fractionation, while for top-down analyses preceded by RP-HPLC fractionation, the presence of non-pure histone fractions may have a significant effect. Nevertheless, obtaining high yield of histone preparation using primary cells remains a challenge. For collection of the data shown here, histones were isolated using the Qiagen Qproteome Nuclear Protein Kit.

### Enzymatic fragmentation of histones and sequence coverage

Mixtures of isolated histones are commonly fractionated by either RP-HPLC or 1-DE [28–30]. The first method yields histones in solution, which can be used for top-down proteomic analyses, while 1-DE fractionation implies in-gel enzymatic digestion for bottom-up experiments. If the latter method is used, the choice of enzyme for histone digestion may have a profound effect. In this study, we used trypsin, which is also used in a vast majority of other proteomic experiments. Because histones are basic proteins with high contents of Lys and Arg residues, the use of trypsin specific for peptide bonds C-terminal to Lys and Arg (Lys-Pro and Arg-Pro bonds are cleaved at very low rate) comes with the disadvantage of cutting histones into short fragments of single amino acids, di-, tri- and tetra-peptides. Such small peptides are excluded from MS/MS analysis because molecular species below 600 Da (or m/z 300^+2^) are not fragmented. On the other hand, if Lys or Arg residues are acetylated or tri-methylated, the C-terminal peptide bond is usually not cleaved by trypsin. Our results (Table 2) show that while using native histone preparations and trypsin digestion we were able to obtain 50 % sequence coverage for Histone H2A, 91 % for Histone H2B, 61 % for Histone H3 and 81 % for Histone H4, and we identified most of the PTMs reported based on analyses of preparations obtained from cell lines. Use of other enzymes, such as chymotrypsin, Arg-C, Glu-C, Asp-N [31] or other proteolytic enzymes might provide more complete coverage by complementing those sequences obtained using trypsin. Our previous work evaluated use of chymotrypsin for histone analysis to obtain almost complete sequence coverage; however, in that study we used recombinant, non-modified histone [28]. Although the extent of total sequence coverage of histones is important, it is equally important to consider which potentially modified residues were missed in our mass spectrometric analysis. A comprehensive overview of sequence coverage and identifications of PTMs resulting from this study is presented in Table 2. The amino acids highlighted in yellow are those that have been found to be modified in this study, while those highlighted in green have been reported as modified in other systems combined per literature reports, but were not found in this study. Segments of each histone sequence that have been identified by mass spectrometry in our study are underlined. Further analysis of data provided in Table 2 shows that due to limitations of our methodological approach, trypsin cuts some regions into very small fragments due to the high concentration of Lys and Arg residues. As a result, we could potentially miss several modifications, including the following: H2BK30, H3K56, H4S1, H4R3, H4K20 and five modifications in the first eight N-terminally located residues in histone H3.

**Table 2:** Summary of PTM identification and sequence coverage of Histones H2A, H2B, H3 and H4 based on combined mass spectrometric analyses^a^

	

^a^The yellow highlighted amino acids we identified in this study as being modified. The green highlighted amino acids are reported in other literature and not found in this study. The underlined sequences are what we identified in this study

### Mass spectrometry profiling of PTMs

Tables 3, 4, 5 and 6 summarize PTMs identified for Histone H2A, H2B, H3 and H4 during the course of this study. These modifications are: acetylation (+42.01), mono-, di- tri-methylation (+14.02, +28.03, +42.05), phosphorylation (+79.98), citrullination (+0.98) and ubiquitination (+114.04). We did not find biotinylation and ADP-ribosylation, while several peptides were identified with oxidated methionine. To exclude the effect of artificial methylation during gel manipulation using acetic acid and methanol for gel de-staining [32], we performed side-by-side comparison by replacing methanol with ethanol in our de-staining protocol. Methylated sites identified in histones from gels exposed to methanol treatment but not identified in histones exposed to ethanol treatment were removed as methodological artifacts.

**Table 3:** PTMs found in Histone H2A of resting macrophage^a^

	LTQ Orbitrap		5600 TripleTof
	CID	ETD	CID
^1^AGGK(+42.01)AGK(+42.01)DSGK(+42.01)AK^13^	+		+
^20^SSRAGLQFPVGR^31^	+		
^23^AGLQFPVGR^31^	+	+	+
^85^HLQLAIR^91^	+	+	+
^85^HLQLAVR(+14.02)^91^	+		
^92^NDEELNK^98^			+
^92^NDEELNKLLGK^102^	+	+	+
^92^NDEELNKLLGR^102^	+		
^92^GDEELDSLIK^101^	+	+	+
^102^ATIAGGGVIPHIHK^115^	+	+	+
^102^ATIAGGGAIPHIHK(+28.03)^115^	+		
^102^VTIAQGGVLPNIQAVLLPK^120^	+	+	+
^102^VTIAQGGVLPNIQAVLLPK(+114.04)K^121^		+	

^a^Acetylation: +42.01; Methylation: +14.02; Di-methylation: +28.03; Ubiquitination: +114.04

**Table 4:** PTMs found in Histone H2B of resting macrophage^a^

	LTQ Orbitrap		5600 TripleTof
	CID	ETD	CID
^1^PEPAK(+42.01)SAPAPK^11^	+		
^1^PDPAK(+42.01)SAPAPK^11^			+
^12^K(+42.01)GSK(+42.01)K(+42.01)AVTK^20^	+		+
^16^K(+42.01)AVTK(+42.01)AQK^23^			+
^34^KESYSVYVYK^43^	+	+	
^34^KESYSIYVYK^43^	+	+	+
^34^K(+42.01)ESYSVYVYK^43^	+	+	+
^34^K(+42.01)ESYSIYVYK^43^	+		+
^35^ESYSVYVYK^43^	+	+	+
^35^ESYSIYVYK^43^	+	+	+
^44^VLKQVHPDTGISSK^57^	+	+	+
^47^QVHPDTGISSK^57^	+	+	+
^58^AMGIMNSFVNDIFER^72^	+	+	+
^58^AMGIMNSFVNDIFER^72^	+	+	+
^73^IAGEASR(+0.98)LAHYNK^85^			+
^87^STITSREIQTAVR^99^	+	+	
^93^EIQTAVR^99^	+	+	+
^100^LLLPGELAK^108^	+	+	+
^109^HAVSEGTKAVTKYTSAK^125^	+	+	+

^a^Acetylation: +42.01; Citrullination: +0.98; M: oxidated methionine

**Table 5:** PTMs found in Histone H3 of resting macrophage^a^

	LTQ Orbitrap		5600 TripleTof
	CID	ETD	CID
^9^K(+42.01)STGGK(+42.01)APR^17^			+
^18^K(+42.01)QLATK(+42.01)AAR^26^	+		+
^18^KQLATK(+42.01)AAR^26^			+
^19^QLATK(+28.08)^23^			+
^19^QLATK(+42.01)AAR^26^			+
^27^K(+42.01)SAPATGGVK^36^			+
^27^K(+42.05)SAPATGGVK^36^	+		+
^27^K(+42.05)SAPATGGVK(+14.02)^36^	+		
^27^K(+42.01)S(+79.97)APATGGVK(+28.03)^36^			+
^41^YRPGTVALR^49^	+	+	+
^57^STELLIR^63^	+	+	+
^64^KLPFQR^69^	+	+	
^65^LPFQR^69^	+	+	
^70^LVR(+0.98)EIAQDFK^79^			+
^73^EIAQDFK^79^	+	+	+
^73^EIAQDFK(+14.02)^79^	+		+
^73^EIAQDFKTDLR^83^	+		
^73^EIAQDFNTDLR^83^	+		+
^73^EIAQDFK(+14.02)TDLR^83^	+	+	+
^73^EIAQDFK(+28.03)TDLR^83^	+	+	
^116^RVTIMPK^122^	+		+
^116^R(+0.98)VTIMPK^122^			+
^116^RVTIMPK^122^	+		+
^117^VTIMPK^122^	+		
^117^VTIMPK^122^	+		
^117^VTIMPKDIQLAR^128^			+
^117^VTIMPKDIQLAR^128^	+		

^a^Acetylation: +42.01; Methylation: +14.02; Di-methylation: +28.03; Tri-methylation: +42.05; Phosphorylation: +79.97; Citrullination: +0.98; M: oxidated methionine

**Table 6:** PTMs found in Histone H4 of resting macrophage^a^

	LTQ Orbitrap		5600 TripleTof
	CID	ETD	CID
^4^GK(+42.01)GGK(+42.01)GLGK(+42.01)GGAK^16^	+		+
^4^GK(+42.01)GGK(+42.01)GLGK^12^			+
^6^GGK(+42.01)GLGK(+42.01)GGAK(+42.01)R^17^	+	+	+
^6^GGK(+42.01)GLGK(+42.01)GGAK^16^	+		+
^9^GLGK(+42.01)GGAK(+42.01)R^17^	+		+
^21^VLR(+0.98)DNIQGITK^31^			+
^24^DNIQGITKPAIR^35^	+	+	+
^24^DNIQGITKPAIRR^36^	+		
^24^DNIQGITK(+42.01)PAIR^35^	+		+
^45^RISGLIYEETR^54^	+	+	+
^45^R(+0.98)ISGLIYEETR^55^			+
^46^ISGLIYEETR^54^	+	+	+
^46^ISGLIYEETR(+14.02)^54^	+	+	
^46^ISGLIYEET(+114.04)R(+28.03)^54^		+	
^60^VFLENVIR^67^	+	+	+
^68^DAVTYTEHAK^77^	+	+	+
^68^DAVTYTEH(+14.02)AK^77^			+
^68^DAVTYTEHAKR^78^		+	+
^79^KTVTAMDVVYALK^91^	+		+
^79^KTVTAMDVVYALK^91^	+	+	+
^79^K(+42.01)TVTAMDVVYALK^91^	+		
^80^TVTAMDVVYALK^91^	+	+	+
^80^TVTAMDVVYALK^91^	+	+	+
^80^TVTAMDVVYALKR^92^			+
^80^TVTAMDVVYALKR^92^	+	+	+
^93^QGRTLYGFGG^102^	+		+
^96^TLYGFGG^102^	+		+

^a^Acetylation: +42.01; Methylation: +14.02; Di-methylation: +28.03; Citrullination: +0.98; Ubiquitination: +114.04; M: oxidated methionine

The next question we wanted to address was whether PTMs reported in the literature and not found in our study were missed due to an insufficient quantity of histones to detect minimally present  modifications or if they do not exist in these cells at their resting stage. For these MS analyses we used 5 μg of trypsin digested histones, although, we also performed one analysis using 15 μg of histone preparation. As expected, an increased amount of histone preparations used for analysis resulted in an increased number of recorded spectra for already identified peptides and their modifications. For example, for ^24^DNIQGITKPAIR^35^ from Histone H4 we recorded 148 spectra when using 5 μg of preparation and 238 spectra when 15 μg of protein were used. For the same peptide with modification H4K31ac, the number of recorded spectra increased from 5 to 12, respectively. Such an increase of material used for mass spectrometry analyses allowed us to identify only one more modification, which was H2A98Kac in the ^92^NDEELNKLLGK^102^ peptide (Table 3), while we identified the non-acetylated form of this peptide using both amounts of histone preparation. Based on this result we conclude that enrichment of tryptic digests of histones and/or using more material for MS analysis may potentially reveal additional PTMs, however we expect that there will be very few of them and at a very low level of abundance in resting macrophages. This situation may change when macrophages become activated with highly elevated cellular activity. Interestingly, we have found only one Ser residue that was phosphorylated (H3S28ph; Table 5) despite the higher extent of phosphorylated histones reported in the literature. Simply increasing the amount of material for MS analyses did not reveal more phosphorylated sites. If such sites do exist, their detection will certainly require sample enrichment using an enrichment method, such as TiO_2_ columns [25].

Primary cells, in this case human MDM, are isolated from various donors (individuals) and are characterized by donor-to-donor variation including allelic polymorphism. Therefore, the obtained data needs extra caution in interpretation, but also may provide additional information. For example, in Histone H2B (Table 4) we have identified ^34^KESYSVYVYK^43^ peptide and its acetylated form ^34^K(ac)ESYSVYVYK^43^ in all three donors. Interestingly, we have also found ^34^KESYSIYVYK^43^ peptide with Val to Ile substitution and its acetylation form ^34^K(ac)ESYSIYVYK^43^. In this case, all three donors have the same Val to Ile substitution, but it might not be the case if cells from other donors are used. This latter finding shows that the epigenetic effect of allelic polymorphism, which has been well documented [33, 34], needs to be considered in data interpretation if such polymorphism affects residues that can be potentially modified.

Although our experimental data showed a degree of conserved acetylated and methylated Lys residues in histones, many studies involving histone PTMs have been done to date using transformed cell lines such as Jurkat, U937, HeLa etc. [31, 35, 36] and little has been done using primary human cells. Although human transformed cell lines reflect mechanisms operative in primary human cells, substantial effort is required to prove experimentally the degree of histone code conservation between transformed and primary cells. An advantage of using cell lines is an almost unlimited amount of material that can be obtained for experiments, while the number of primary cells from donors is definitively limited. In addition, primary cells obtained from various donors will certainly represent inherent variability existing within any population of individuals, while cell lines typically represent one individual who served as a donor, very often in the distant past such as in the case of HeLa cells [37]. Regardless of known drawbacks, MDMs are still an attractive primary cell model as they reflect the *in vivo* response much closer than transformed cell lines.

**Fig. 1:**
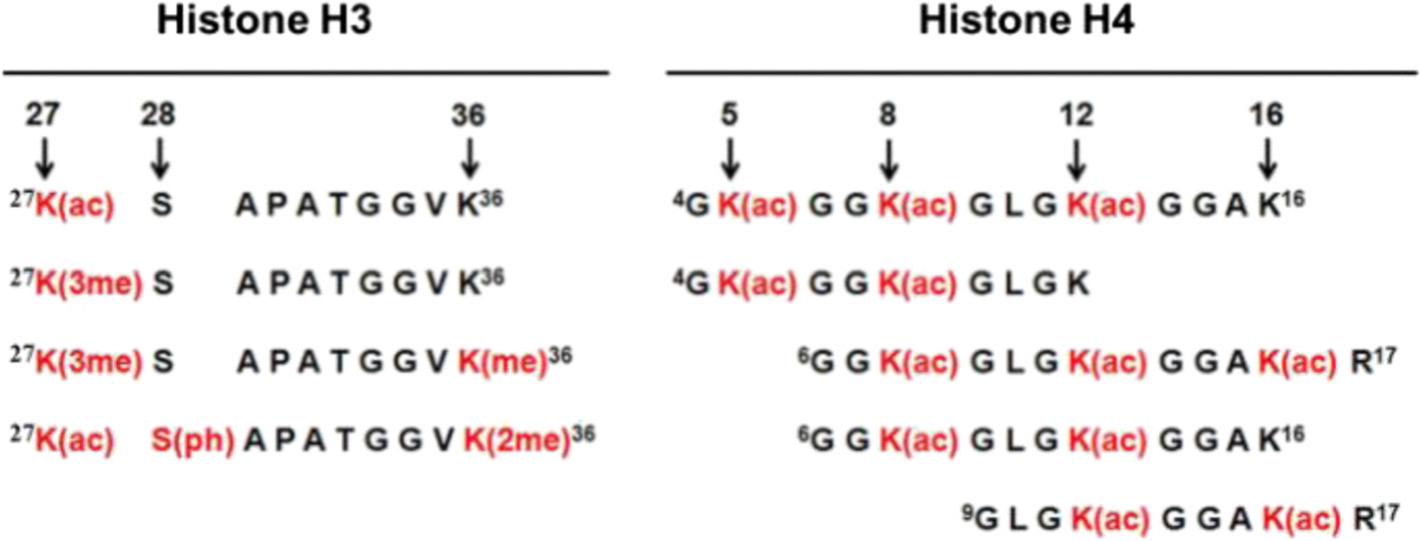
Variability of PTM patterns in N-terminal regions of histones H3 and H4. Although some modifications are shared by more than one pattern, each of them likely represents different regulatory mechanisms

Another advantage of mass spectrometric profiling of histone PTMs is that despite sharing specific modification(s), the overall modification pattern of a specific histone region might be different, thus its regulatory effect may vary. Figure 1 shows an example of such pattern differences for N-terminal tails of histones H3 and H4. Although enzymes may modify many of the same residues on histones, the substrate specificity or selectivity for modifications at particular sites is not clear. Moreover, it is not clear whether patterns presented in Fig. 1 result in differential modifications or differential removal of specific groups. Addressing the order and/or dynamic of histone PTMs would require various experimental approaches including measurements of activity and cellular location of modifying enzymes [38] leading to defining the role of such distinct patterns in transcriptional regulation [39]. To further complicate matters, it is possible that the modification of one site is dependent on the modification or lack of modification of another site, as has been shown for ubiquitination of the H2BK120ub residue and tri-methylation of the H3K49(me3) and bH3K79(me3) residues [19]. This gives evidence that PTM cross-talk is emerging and gaining importance in the interpretation of regulatory effects.

The majority of the data generated from this study was provided by a combination of qTof/Triple TOF (ABSciex) and LTQ XL Orbitrap with CID (Thermo) systems, except for ubiquitination, which was identified only using LTQ XL Orbitrap with ETD method. The latter could be due to the fragile nature of ubiquitination and the ETD providing a gentler fragmentation, whereas CID fragmentation in both the qTOF and Orbitrap is more energetic, producing more signature transitions for overall identifications of modified peptides. Therefore, it is beneficial to use complementary mass spectrometry platforms and methods of data acquisition to increase sequence and PTM coverage. As dependent as identification of acetylation and methylation is on the amount of material, we expect sample enrichment techniques such as TiO_2_ columns/cartridges to broaden identification of phosphorylated peptides. Immunoprecipitation using a specific antibody will apply to the detection of ubiquitinated peptides.

**Fig. 2:**
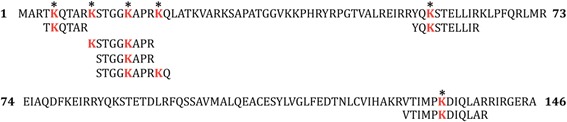
Propionylation. Identification of propionylated Lys residues (marked with *) in histone H3. All propionylated sites were found in histone preparations from resting MDMs of four donors

C-terminal peptide bonds of lysine residues are cleaved by trypsin unless ε-amine is blocked by acetylation. Therefore, unmodified histones rich in lysine residues are fragmented into short peptides. Propionylation of ε-amines prevents trypsin from cleaving these peptide bonds and allows non-modified Lys and to be identified by mass spectrometry. In this study, we performed propionylation of Histone H3 before and after trypsin digestion, followed by mass spectrometry analysis. We found peptides with propionylated Lys residues at positions 4, 9, 14 and 18 (Fig. 2). This result combined with detecting acetylated Lys residues shows that Lys at position 4 is indeed not modified in resting primary macrophages and that some Lys residues at positions 9, 14 and 18 are not acetylated, thus contributing to the complex mosaic of PTMs in this region. The exact ratio of acetylation of Lys residues in this particular region of Histone H3 will be subjected to a separate and more focused study.

### Histidine (His) methylation

**Fig. 3:**
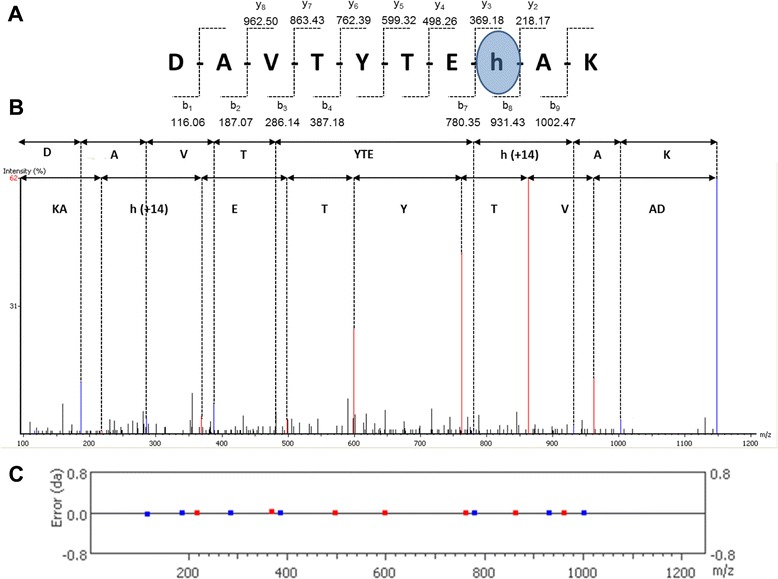
Identification of His monomethylation. MS/MS spectrum of Histone H4 peptides marked with mono-methylated histidine acquired using qTOF. **a** Peptide sequence and assignment of y and b transitions; **b** Spectrum with corresponding assignment of amino acids; **c** Mass error for fragment ions assignment

Herein, we report, for the first time, methylation of His at position 75 in Histone H4 in primary human MDM. Figure 3 shows mass spectrum of ^68^DAVTYTEH(+14.02)AK^77^ modified by adding mass of 14 Da to His. Identification of this PTM was detected using qTOF (AB Sciex 5600 TripleTof) with all transitions lined-up without significant deviation from their theoretical (expected) mass.

In 1972, Borun and colleagues demonstrated that His is methylated in histones during the HeLa S-3 cell cycle [40]. Since then, His methylation has been found in a variety of proteins [41–44], however, for many years, protein methylation was outside mainstream research interests, mostly because of its unclear role in biological processes. Since the mid-1990s, Lys and Arg methylation have gained significant attention as a result of general progress in research describing the biological importance of this specific PTM [45]. Nevertheless, the role of His methylation in the regulation of biological processes is lagging and a relatively small number of studies have been published. Boffa et al. reported that carcinogens, such as alkylating carcinogen 1,2-dimethylhydrazine (DMH), induces colonic tumors and leads to non-random methylation of His in Histone H1, which is not normally seen in this protein [46, 47]. The role of H4H75me1 in epigenetic regulation is intriguing and we are pursuing it in subsequent studies, particularly because in a concurrent study we have documented the novel observation of a significant decrease of this modification when MDMs were exposed to chemical stress (data not shown here).

### Ubiquitination and citrullination

**Fig. 4:**
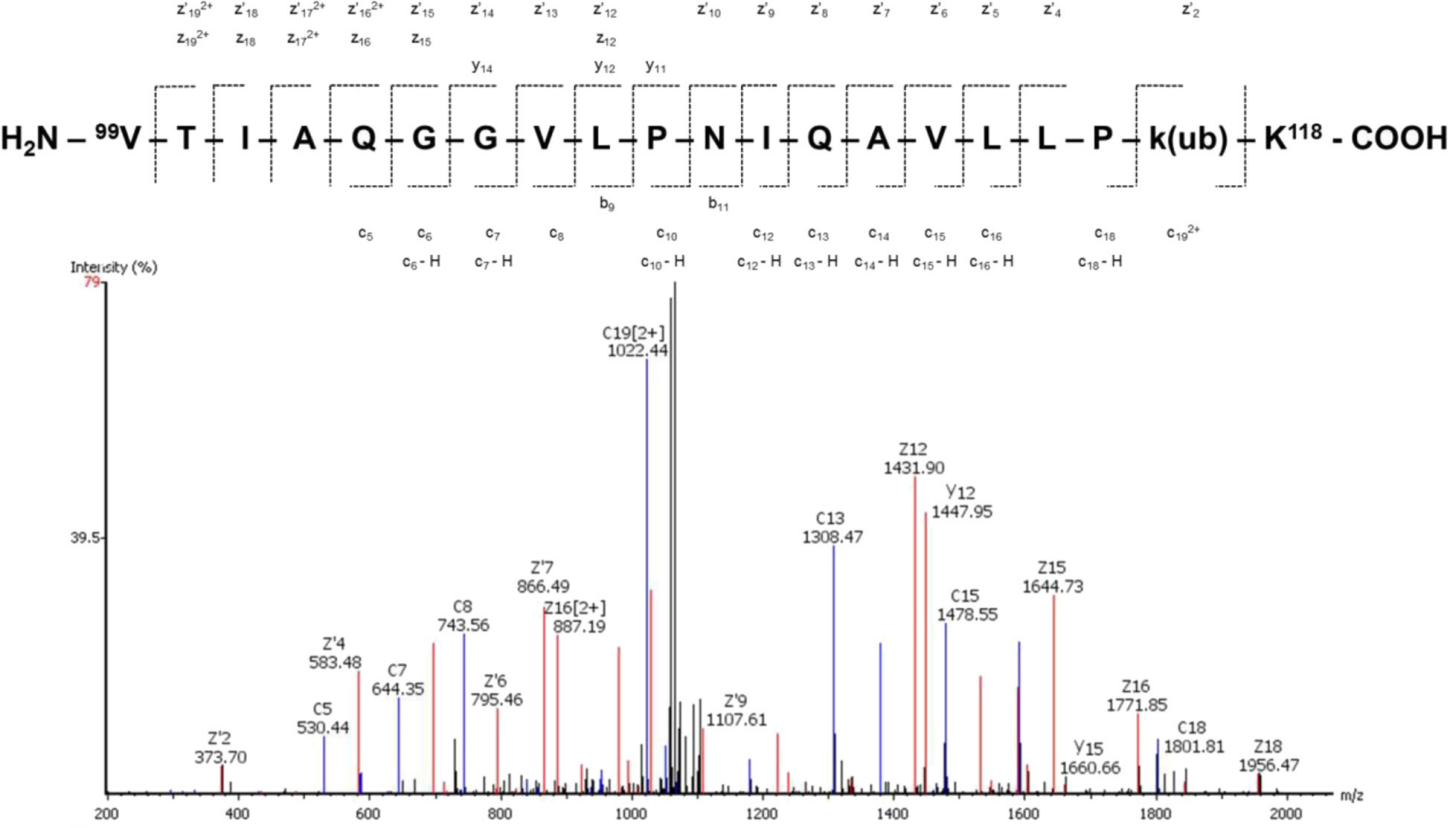
Identification of Lys ubiquitination. MS/MS spectrum of peptide from Histone H2A (H2AV, Q71UI9) peptide with ubiquitinated Lys at position 120 acquired using LTQ Orbitrap with ETD fragmentation. Peptide sequence and fragment ions assignment is shown above the spectrum

A major advantage of the ETD method of data acquisition is the ability to identify ubiquitination. In this study, we found H2AK120 ubiquitinated (H2AV isoform, UniProt# Q71UI9) and the corresponding mass spectrum is shown in Fig. 4. The position of the ubiquitinated Lys residue is always the first in a doublet of Lys-Lys di-peptides; however, its numerical position may be different depending on the isoform (see Additional file 1: Table S1) specific to a particular donor. Nevertheless, this ubiquitinated Lys corresponds to H2AK119, which is found in other cells such as yeast or the RAW264.7 mouse monocytes/macrophage cell line [48]. Ubiquitination of H2AK120 is commonly observed [49] and it has also been shown to be associated with transcriptional repression of chemokines or chemokine ligands such as CCL5, CXCL10 and CXCL2, however not CXCL1 [48]. Another ubiquitinated site found in our study was H4T54ub, which was detected in conjunction with H4R55me2 modification and assignment of transitions from ETD MS/MS reported high confidence. Our mass spectrometric analysis did not confirm the presence of H2BK120ub, which was found in the U937 human leukemia cell line [50].

Citrullination is an enzymatic deamination of protein bound arginine by peptidylarginine deiminases (PAD) and is considered as a PTM with an additional mass of 0.98 Da. We have identified the following five peptides with citrullination (arginine deimination) modifications: 1) ^73^IAGEASR(+0.98)LAHYNK^85^ from Histone H2B, 2) ^70^LVR(+0.98)EIAQDFK^79^, 3) ^116^R(+0.98)VTIMPK^122^ from Histone H3, 4) ^21^VLR(+0.98)DNIQGITK^31^, and 5) ^45^R(+0.98)ISGLIYEETR^55^ from Histone H4. Dimethylation prevents Arg from being deiminated by PAD, however, mono-methylated Arg can be deiminated and converted to citrullin [51]. Because mono-methylation is associated with transcription activation, citrullination preventing Arg methylation is considered as a transcription repressor.

## Conclusions

This study is the first comprehensive profiling of histone PTMs in primary human MDM. Monocytes were grown in the presence of MCSF to support differentiation to macrophages. Although this could be considered as stimulation, we consider this state of MDMs as resting and any subsequent activation would be considered as a response to external stimuli such as cytokines, microbial products, viral infection and inflammation. Although MDMs maintain their metabolism, we may expect relatively low levels of PTMs to promote transcription and relatively high levels of PTMs responsible for transcription suppression. In support of this conclusion, we observed as many as five Arg residues being deimidated (converted to citrullin) in Histones H2B, H3 and H4, which are considered transcription repressors.

Some regions of histones, i.e., N-terminal tail of H3, contain multiple Lys and Arg residues either next to each other or apart by one or two other amino acids. When digested with trypsin, they yield very short peptides or single amino acids, which are not recorded using the standard data dependent acquisition mass spectrometry method. One exception is the acetylation of Lys, which protects the peptide bond from trypsin digestion. In these situations, specific antibodies have an edge in detecting changes.

Methylation of His residues in histones is rarely observed; therefore, our identification of methylated His at position 75 in Histone H4 can be considered quite novel. Potential regulatory functions of this modification remain highly speculative, yet intriguing, and will require future investigations. Further studies of this histone modification are warranted and will shed new light on how it may contribute to the epigenetic regulation of resting and activated macrophage.

## Additional file

Additional file 1: Table S1. Summary of variants and isoforms of human histones. (DOCX 33 kb)
