# Supplementary material for: Profiling post-translational modifications of histones in human monocyte-derived macrophages
Source: Proteome Sci. 2015 Sep 24;13:24. doi: 10.1186/s12953-015-0080-7 (PMC4582717; doi:10.1186/s12953-015-0080-7)
Supplement: Additional file 1: Table S1. — Summary of variants and isoforms of human histones. (DOCX 33 kb) [file 12953_2015_80_MOESM1_ESM.docx]

Additional file 1: Table S1. Summary of variants and isoforms of human histones

| **Histone H2A** | **Isoform** | **Accession number** | **Length (a.a.)** | **Mutations** |
| --- | --- | --- | --- | --- |
| H2AV |  | Q71UI9 |  |  |
|  | 1 isoform | Q71UI9-1 | 127 | ‘canonical’ sequence |
|  | 2 isoform | Q71UI9-2 | 113 | [108-12](http://www.uniprot.org/blast/?about=Q71UI9%5b109-128%5d)7: GVIPHIHKSLIGKKGQQKTA → EKRRCS |
|  | 3 isoform | Q71UI9-3 | 89 | [27-6](http://www.uniprot.org/blast/?about=Q71UI9%5b28-65%5d)4: Missing |
|  | 4 isoform | Q71UI9-4 | 101 | [1-2](http://www.uniprot.org/blast/?about=Q71UI9%5b1-27%5d)7: AGGKAGKDSGKAKAKAVSRSQRAGLQ → F |
| H2AZ | ---------- | P0C0S5 | 127 | 14: A → T  38: T → S  127: A → V |
| H2AX | ---------- | P16104 | 142 | 1-5: AGGKA → SGR  8-9: DS → TG  13: K → R  17: V → K  21: Q → S  32: I → V  35: H → L  37-44: KTRTTSHG → RKGHYAE  49: T → G  51: A → P  54: S → L  57: I → V  65: V → I  73-74: SD → AR  76: L → N  78-79: VK → KT  82: T → I  92: G → N  97-98: DS → NK  100-102: IKA → LGGV  106: G → Q  110: I → L  112: H → N  114-116: HKS → GAV  118-119: IG → LP  122-127: GQQKTA → TSATVGPKAPSGGKKATQASQEY |
| H2A1H | ---------- | Q96KK5 | 127 | 1-3: AGG → SGRG  5: A → Q  7-10: KDSG → G  13: K → R  17-18: VS → KT  21: Q → S  32: I → V  35: H → L  37-43: KTRTTSH → RK  44-45: GR → GNYAER  59: T → G  51: A → P  54: S → L  57: I → V  65: V → I  73-74: SK → AR  76: L → N  78-79: VK → KT  82: T → I  92: G → N  97-98: DS → NK  100: I → LG  102: A → V  106: G → Q  110: I → L  112: H → N  114-116: HKS → QAV  118-119: IG → LP  122-127: GQQKTA → TESHHKAK |
| H2A1 | ---------- | P0C0S8 | 129 | 1: A → S  2: G → GR  5: A → Q  7-10: KDSG → G  13: K → R  17-18: VS → KT  21: Q → S  32: I → V  35: H → L  37-43: KTRTTSH → RK  44: G → GNYAE  49: T → G  51: A → P  54: S → L  57: I → V  65: V → I  73-74: SK → AR  76: L → N  78-79: VK → KT  82: T → I  92: G → N  97-98: DS → NK  100: I → LG  102: A → V  106: G → Q  110: I → L  112: H → N  114-116: HKS → QAV  118-119: IG → LP  122-127: GQQKTA → TESHHKAKGK |
| H2A1D | ---------- | P20671 | 129 | 1: A → S  2: G → GR  5: A → Q  7-10: KDSG → G  13: K → R  17-18: VS → KT  21: Q → S  32: I → V  35: H → L  37-43: KTRTTSH → RK  44: G → GNYSE  49: T → G  51: A → P  54: S → L  57: I → V  65: V → I  73-74: SK → AR  76: L → N  78-79: VK → KT  82: T → I  92: G → N  97-98: DS → NK  100: I → LG  102: A → V  106: G → Q  110: I → L  112: H → N  114-116: HKS → QAV  118-119: IG → LP  122-127: GQQKTA → TESHHKAKGK |
| H2A1J | ---------- | Q99878 | 127 | 1: A → S  2: G → GR  5: A → Q  7-10: KDSG → G  13: K → R  17-18: VS → KT  21: Q → S  32: I → V  35: H → L  37-43: KTRTTSH → RK  44: G → GNYAE  49: T → G  51: A → P  54: S → L  57: I → V  65: V → I  73-74: SK → AR  76: L → N  78-79: VK → KT  82: T → I  92: G → N  97-98: DS → NK  100: I → LG  102: A → V  106: G → Q  110: I → L  102: H → N  114-116: HKS → QAV  118-119: IG → LP  122-124: GQQ → TESHH  127: A → K |
| H2A2A | ---------- | Q6FI13 | 129 | 1: A → S  2: G → GR  5: A → Q  7-10: KDSG → G  13: K → R  17: V → K  21: Q → S  32: I → V  35: H → L  37-43: KTRTTSH → RK  44: G → GNYAE  49: T → G  51: A → P  54: S → M  57: I → V  65: V → I  73-74: SK → AR  76: L → N  78-79: VK → KT  82: T → I  92: G → N  97-98: DS → NK  100: I → LG  102: A → V  106: G → Q  110: I → L  112: H → N  114-116: HKS → QAV  118-119: IG → LP  122-127: GQQKTA → TESHHKAKGK |
| H2A2C | ---------- | Q16777 | 128 | 1: A → S  2: G → GR  5: A → Q  7-10: KDSG → G  13: K → R  17: V → K  21: Q → S  32: I → V  35: H → L  37-43: KTRTTSH → RK  44: G → GNYAE  49: T → G  51: A → P  54: S → M  57: I → V  65: V → I  73-74: SK → AR  76: L → N  78-79: VK → KT  82: T → I  92: G → N  97-98: DS → NK  100: I → LG  102: A → V  106: G → Q  110: I → L  112: H → N  114-116: HKS → QAV  118-119: IG → LP  122-127: GQQKTA → TESHKAKSK |
| H2AJ |  | Q9BTM1 |  |  |
|  | 1 isoform | Q9BTM1-1 | 128 | 1: A → S  2: G → GR  5: A → Q  7-10: KDSG → G  12-13: AK → VR  17: V → K  21: Q → S  32: I → V  35: H → L  37-43: KTRTTSH → RK  44: G → GNYAE  49: T → G  51: A → P  54: S → L  57: I → V  65: V → I  73-74: SK → AR  76: L → N  78-79: VK → KT  82: T → I  92: G → N  97-98: DS → NK  100: I → LG  102: A → V  106: G → Q  110: I → L  112: H → N  114-116: HKS → QAV  118-119: IG → LP  122-123: GQ → TES  127: A → KSK |
|  | 2 isoform | Q9BTM1-2 | 150 | 1: A → S  2: G → GR  5: A → Q  7-10: KDSG → G  12-13: AK → VR  17: V → K  21: Q → S  32: I → V  35: H → L  37-43: KTRTTSH → RK  44: G → GNYAE  49: T → G  51: A → P  54: S → L  57: I → V  65: V → I  73-74: SK → AR  76: L → N  78-79: VK → KT  82: T → I  92: G → N  97-98: DS → NK  100: I → LG  102: A → V  106: G → Q  110: I → L  112: H → N  114-116: HKS → QAV  118-119: IG → LP  120-127: KKGQQKTA → VCEHSGPSSGKIPSDRAELGAGSVCGHIFQKVE |
| H2A3 | ---------- | Q7L7L0 | 129 | 1: A → S  2: G → GR  5: A → Q  7-10: KDSG → G  13: K → R  17: V → K  21: Q → S  32: I → V  35: H → L  37-38: KT → RK  39-44: RTTSHG → G  45: R → NYSER  51: A → P  54: S → L  57: I → V  65: V → I  73-74: SK → AR  76: L → N  78-79: VK → KT  82: T → I  92: G → N  97-98: DS → NK  100: I → LG  101-102: KA → RV  106: G → Q  110: I → L  112: H → N  114-116: HKS → QAV  118-119: IG → LP  122-127: GQQKTA → TESHHKAKGK |
|  |  |  |  |  |
| **Histone H2B** | **Isoform** | **Accession number** | **Length (a.a.)** | **Mutations** |
| H2B1B |  | P33778 | 125 | ‘canonical’ sequence |
| H2B3B |  | Q8N257 | 125 | 2: E → D  18: I → V  32: S → G  75: G → S  94: I → V |
| H2B1C |  | P62807 | 125 | 4: S → A  18: I → V  39: I → V |
| H2B1D |  | P58876 | 125 | 4: S → T  18: I → V  39: I → V |
| H2B2E |  | Q16778 | 125 | 4: S → A  18: I → V |
| H2B1F |  | P10853 | 125 | 4: S → A  18: I → V  39: I → V  75: G → S |
| H2B2F |  | Q5QNW6 | 125 | 2: E → D  4: S → A  18: I → V  21: A → V  39: I → V |
| H2BFS |  | P57053 | 125 | 4: S → A  18: I → V  27: K → R  39: I → V  81: A → P  124: S → A |
| H2B1H |  | Q93079 | 125 | 2: E → D  4: S → A  18: I → V  39: I → V |
| H2B1J |  | P06899 | 125 | 4: S → A  18: I → V  124: S → A |
| H2B1K |  | O60814 | 125 | 4: S → A  18: I → V  39: I → V  124: S → A |
| H2B1L |  | Q99880 | 125 | 3-4: PS → LA  18: I → V  39: I → V  75: G → S |
| H2B1M |  | Q99879 | 125 | 4: S → V  9: A → V  19: T → N  39: I → V |
| H2B1N |  | Q99877 | 125 | 18: I → V  39: I → V |
| H2B1O |  | P23527 | 125 | 2: E → D  4: S → A  18: I → V |
|  |  |  |  |  |
| **Histone H3** | **Isoform** | **Accession number** | **Length (a.a.)** | **Mutations** |
| H3.1 |  | P68431 | 135 | ‘canonical’ sequence |
| H3.1T |  | Q16695 | 135 | 24: A → V  71: V → M  98: A → S  111: A → V |
| H3.2 |  | Q71DI3 | 135 | 96: C → S |
| H3.3 |  | P84243 | 135 | 31: A → S  87: S → A  89-100: VM → IG  96: C → S |
| H3.5 |  | Q6NXT2 | 134 | 29: A → T  31: A → S  33: G → C  37-38: KP → P  79: K → N  87: S → A  90: M → G  96: C → S  104: F → L |
|  |  |  |  |  |
| **Histone H4** | **Isoform** | **Accession number** | **Length (a.a.)** | **Mutations** |
| H4 |  | P62805 | 102 | ---------------------------------------- |
